# Development of a Novel Anti-CD19 Chimeric Antigen Receptor: A Paradigm for an Affordable CAR T Cell Production at Academic Institutions

**DOI:** 10.1016/j.omtm.2018.11.010

**Published:** 2018-12-06

**Authors:** Maria Castella, Anna Boronat, Raquel Martín-Ibáñez, Vanina Rodríguez, Guillermo Suñé, Miguel Caballero, Berta Marzal, Lorena Pérez-Amill, Beatriz Martín-Antonio, Julio Castaño, Clara Bueno, Olga Balagué, Europa Azucena González-Navarro, Carles Serra-Pages, Pablo Engel, Ramon Vilella, Daniel Benitez-Ribas, Valentín Ortiz-Maldonado, Joan Cid, Jaime Tabera, Josep M. Canals, Miquel Lozano, Tycho Baumann, Anna Vilarrodona, Esteve Trias, Elías Campo, Pablo Menendez, Álvaro Urbano-Ispizua, Jordi Yagüe, Patricia Pérez-Galán, Susana Rives, Julio Delgado, Manel Juan

**Affiliations:** 1 Department of Hematology, ICMHO, Hospital Clínic de Barcelona, Villarroel 170, 08036 Barcelona, Spain; 2 Department of Immunology, CDB, Hospital Clínic de Barcelona, Villarroel 170, 08036 Barcelona, Spain; 3 Institut d’Investigacions Biomèdiques August Pi i Sunyer - IDIBAPS, Rosselló 153, 08036 Barcelona, Spain; 4 Stem Cells and Regenerative Medicine Laboratory, Production and Validation Center of Advanced Therapies (Creatio), Department of Biomedical Sciences, University of Barcelona, Casanova 143, 08036 Barcelona, Spain; 5 Physiopathology and Molecular Bases in Hematology Group, IDIBAPS, Rosselló 153, 08036 Barcelona, Spain; 6 Institut de Recerca Pediàtrica Hospital Sant Joan de Déu, Universidad de Barcelona, Passeig de Sant Joan de Déu, 2, 08950 Esplugues de Llobregat, Barcelona, Spain; 7 Josep Carreras Leukemia Research Institute, Department of Biomedicine, School of Medicine, University of Barcelona, Casanova 143, 08036 Barcelona, Spain; 8 Department of Pathology, Hospital Clínic, IDIBAPS, Villarroel 170, 08036 Barcelona, Spain; 9 Universitat de Barcelona, Casanova 143, 08036 Barcelona, Spain; 10 Department of Hemotherapy and Hemostasis, ICMHO, Hospital Clínic de Barcelona, Villarroel 170, 08036 Barcelona, Spain; 11 Unit of Advanced Therapies, Hospital Clinic de Barcelona, Blood and Tissue Bank -BST-, Passeig del Taulat, 106, 08005 Barcelona, Spain; 12 Centro de Investigación Biomedica en Red de Cancer (ISCIII-CIBERONC), Barcelona, Spain; 13 Institució Catalana de Recerca i Estudis Avancats (ICREA), Barcelona, Spain; 14 Immunology Unit, Department of Biomedical Sciences, University of Barcelona, Casanova 143, 08036 Barcelona, Spain

**Keywords:** chimeric antigen receptor, CD19, leukemia, lymphoma, immunotherapy, 4-1BB, T cell, preclinical studies

## Abstract

Genetically modifying autologous T cells to express an anti-CD19 chimeric antigen receptor (CAR) has shown impressive response rates for the treatment of CD19+ B cell malignancies in several clinical trials (CTs). Making this treatment available to our patients prompted us to develop a novel CART19 based on our own anti-CD19 antibody (A3B1), followed by CD8 hinge and transmembrane region, 4-1BB- and CD3z-signaling domains. We show that A3B1 CAR T cells are highly cytotoxic and specific against CD19+ cells *in vitro*, inducing secretion of pro-inflammatory cytokines and CAR T cell proliferation. *In vivo*, A3B1 CAR T cells are able to fully control disease progression in an NOD.Cg-*Prkdc*^*scid*^*Il2rd*^*tm1Wjl*^/SzJ (NSG) xenograph B-ALL mouse model. Based on the pre-clinical data, we conclude that our CART19 is clearly functional against CD19+ cells, to a level similar to other CAR19s currently being used in the clinic. Concurrently, we describe the implementation of our CAR T cell production system, using lentiviral vector and CliniMACS Prodigy, within a medium-sized academic institution. The results of the validation phase show our system is robust and reproducible, while maintaining a low cost that is affordable for academic institutions. Our model can serve as a paradigm for similar institutions, and it may help to make CAR T cell treatment available to all patients.

## Introduction

Genetically modifying autologous T cells to express chimeric antigen receptors (CARs), thus redirecting them to eliminate tumor cells, is a new and revolutionary therapeutic modality for cancer treatment and, in particular, for CD19+ B cell malignancies.^1^, ^2^, ^3^, ^4^, ^5^, ^6^, ^7^, ^8^, ^9^

CARs are composed of an extracellular region responsible for binding to a particular antigen and an intracellular region that promotes T cell cytotoxic activity and proliferation. CAR binding to the selected antigen is usually mediated by a single-chain variable fragment (scFv) of a monoclonal antibody. The scFv-derived region results in a medium- to high-affinity and major histocompatibility complex (MHC)-independent interaction of the CAR with its ligand. As a second generation CAR, this scFv is combined with an intracellular co-stimulatory domain (usually CD28 or 4-1BB) and a pro-activator cytotoxic domain (CD3z).^10^, ^11^, ^12^

After initial disappointing results with first-generation CARs, the most recent clinical trials with second-generation anti-CD19 CAR T cells have shown remarkable results in patients with chronic lymphocytic leukemia, non-Hodgkin’s lymphoma, and acute lymphoid leukemia (ALL), reviewed in Wang et al.^8^ and Lorentzen and Straten.^13^ Several academic groups, including the University of Pennsylvania, Memorial Sloan Kettering Cancer Center, the National Cancer Institute, and the Fred Hutchinson Cancer Research Center, pioneered these seminal studies using slightly different CAR constructs in terms of ScFv sequence, co-stimulatory domain, and transmembrane-hinge region. These CARs are currently under evaluation in a number of international multi-center clinical trials. Two of them, tisagenlecleucel (Kymriah, Novartis) and axicabtagene ciloleucel (Yescarta, Kyte-Gilead), have been approved by the U.S. Food and Drug Administration, European Union (EU), and Canada for clinical use. Regarding efficacy, response rates range from 50% to 85%, depending on the type of B cell malignancy and CAR construct, with quite remarkable disease-free and overall survival.^13^ In terms of safety, patients who respond to therapy usually develop persistent B cell aplasia and transitory cytokine release syndrome, which could be severe in a small proportion of patients.^14^

Despite these striking results, this therapeutic approach is currently available only in a handful of centers. With Kymriah and Yescarta approval for certain diseases, this treatment will become available in multiple places (in and outside the United States), although the high cost can still limit access. For these reasons, we decided to develop our own CAR construct targeting CD19 (ARI-0001) that comprises an scFv of our own anti-CD19 monoclonal antibody (mAb) (A3B1) plus the 4-1BB- and CD3z-signaling domains. This paper shows the results of pre-clinical work as well as the description of our production system for ARI-0001 cells, focusing on the results of the validation phase of our clinical trial (CART19-BE-01), which has enrolled and treated more than 20 patients and is currently ongoing. Our work demonstrates the feasibility of producing CAR T cells in medium-sized academic institutions.

## Results

### Validation of Anti-hCD19 A3B1 mAb

Our anti-hCD19 A3B1 mAb has been used in previous studies.^15^ Nevertheless, we designed a set of experiments to further confirm its specificity. As shown in Figure S1A, anti-hCD19 A3B1 reacted against the mouse lymphoma cell line 300.19 transfected with hCD19, but not with untransfected cells. Anti-hCD19 A3B1 also reacted with a subset of human peripheral blood cells, as expected (Figure S1B). Anti-hCD19 A3B1 reacted with B cell lines Raji and Daudi, while no reactivity was observed when T, myeloid, or natural killer (NK) cell lines were used, consistent with the pattern of CD19 expression (Figure S1C). Furthermore, we show that pre-incubation of Daudi cells with the anti-CD19 FMC63 antibody blocked the binding of A3B1, confirming its specificity for CD19 (Figure S1D). These data also suggest that A3B1 and FMC63 antibodies have overlapping epitopes. Finally, a band of around 100 kDa was precipitated from Daudi cells using anti-hCD19 A3B1, consistent with the expected molecular weight of CD19 (Figure S1E). All these data together indicate that A3B1 is a highly sensitive and specific antibody for human CD19 protein.

### *In Vitro* Evaluation of ARI-0001 Efficacy

**Figure 1:**
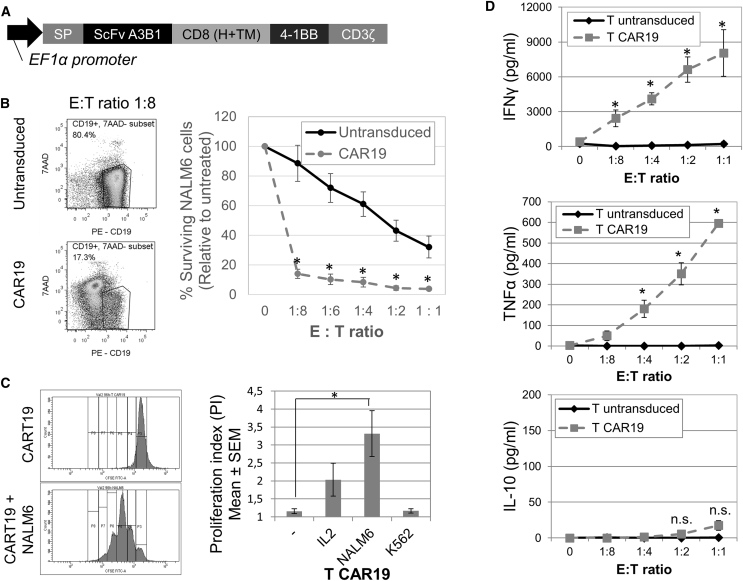
ARI-0001 Anti-tumor Activity *In Vitro* (A) Diagram of A3B1 CAR19 construct. (B) Cytotoxicity assay of CART19 cells versus NALM6 cells at the 16-hr time point. Percent target surviving cells, relative to untreated, is shown (mean of 3 experiments ± SEM). Panels on the right show representative flow cytometry plots at E:T ratio = 1:8. (C) CAR19 T cell proliferation *in vitro* measured by CFSE assay at the 96-hr time point. Panels on the left show representative flow cytometry images. Panel on the right shows quantification of the proliferation index (PI). Mean of 4 experiments ± SEM is shown. (D) Cytokine production (IFNγ, TNF-α, and IL-10) of CART19 cells in co-culture with NALM6 cells at the 16-hr time point, measured by ELISA. Mean of 3 experiments ± SEM is shown. *Statistical significance, p < 0.05; n.s., not statistically significant.

The scFv of anti-CD19 A3B1 antibody was cloned in frame with the rest of the CAR-signaling domains in a lentiviral vector (pCCL) (Figure 1A). For the evaluation of CAR19 A3B1 efficacy, peripheral blood mononuclear cells (PBMCs) isolated from buffy-coats were activated using CD3 and CD28 dynabeads and subsequently transduced using CAR19-containing lentivirus. After an expansion period, the expression of CAR19 on T cells was confirmed by flow cytometry (Figure S2A). The percentage of ARI-0001 cells varied between 20% and 56%, depending on the experiment.

Cytotoxicity of ARI-0001 cells was measured by the *in vitro* eradication of the CD19-positive NALM6 cell line. For this purpose, we developed a flow cytometry-based assay to quantify the number of viable, CD19+ cells (see Materials and Methods and Figure S3). NALM6 cells were almost completely eliminated after 16 hr of co-culture, even after very low effector (E):target (T) ratios (1 effector cell for every 8 target cells). We also observed a minor cytotoxic effect of untransduced (UT) cells due to alloreactivity (Figure 1B). Target cell specificity was also tested by measuring the survival of a CD19-negative HL60 cell line in co-culture with ARI-0001 cells. As expected, no ARI-0001-mediated killing was appreciated in this case (Figure S2B). The cytotoxicity of ARI-0001 cells was also tested against primary B cell acute lymphocytic leukemia (B-ALL) cells, demonstrating similar efficacy (Figure S2C). All these data together indicate that our ARI-0001 cells exhibit a potent and specific cytotoxic effect against CD19-positive cells *in vitro*.

To better characterize ARI-0001 cell response upon CD19 binding, cell proliferation was measured by using a standard carboxyfluorescein diacetate succinimidyl ester (CFSE) assay at the 96-hr time point. Antigen binding should be able to promote ARI-0001 cell expansion in order to eliminate a tumor *in vivo*. As shown in Figure 1C, ARI-0001 cells proliferated in contact with the CD19+ NALM6 cell line and in response to interleukin-2 (IL-2) (to a minor extent). No proliferation was observed in the absence of stimulus or in contact with a CD19-negative cell line (K562), confirming that cell proliferation was mediated by CD19 recognition.

Finally, ARI-0001 cell production of cytokines was measured in the supernatant of effector-target cell co-cultures after 16 hr and analyzed using an ELISA. Cytokine levels from co-cultures using ARI-0001 or UT cells were compared (Figure 1D). While UT cells did not show an increase in interferon (IFN)γ and tumor necrosis factor alpha (TNF-α), ARI-0001 cells showed a significant increase in these two pro-inflammatory cytokines. As expected, a very minor and not significant increase in the anti-inflammatory cytokine IL-10 was observed.

### *In Vivo* Evaluation of ARI-0001 Efficacy

To evaluate the efficacy of the CART19 cells *in vivo*, we performed a xenograft experiment in NOD.Cg-*Prkdc*^*scid*^ *Il2rd*^*tm1Wjl*^/SzJ (NSG) mice.

Mice were randomly allocated to the administration of vehicle (A), UT cells (B), ARI-0001 cells (C), NALM6 cells (D), NALM6 plus UT cells (E), and NALM6 plus ARI-0001 cells (F). Mice corresponding to groups D–F were inoculated with NALM6-Luc + GFP + (CD19+) cells through their tail vein on day 1. On day 4, mice belonging to groups B, C, E, and F were infused with either UT cells or ARI-0001 cells.

**Figure 2:**
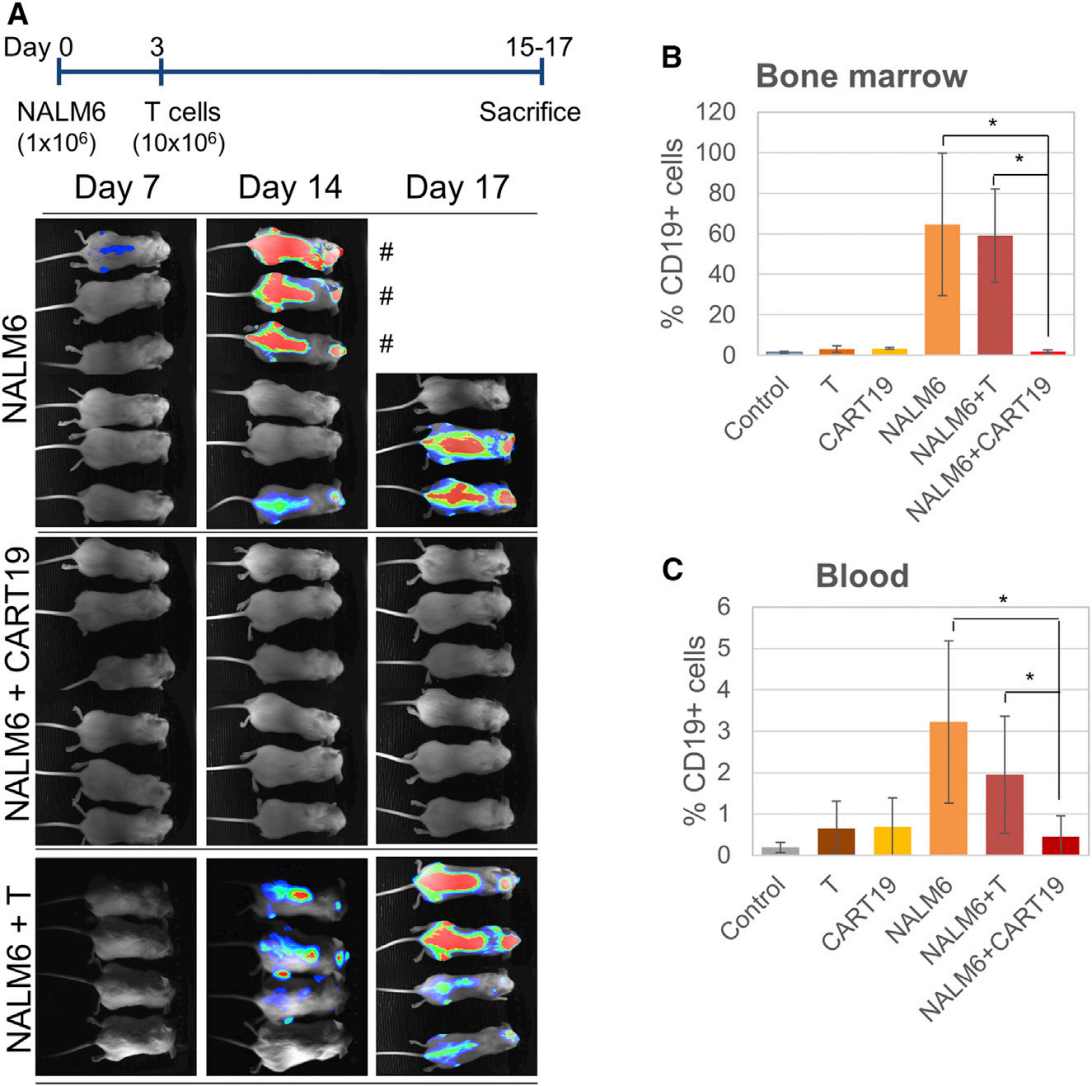
ARI-0001 Anti-tumor Activity *In Vivo* (A) Upper panel shows a timeline of experimental design. Lower panels show bioluminescence images showing disease progression at different days. Animals indicated by a pound sign were sacrificed at day 16 due to advanced disease progression. The rest of the animals were sacrificed at day 17. (B) Detection of tumor (CD19+) cells in the bone marrow of mice shown in (A) (mean ± SD). (C) Detection of tumor (CD19+) cells in blood.

As shown in Figure 2A, disease progression was clearly observed during a period of 2 weeks following NALM6 cell infusion in the NALM6 group (5 of 6 animals progressed) and the NALM6 + UT cell group (4 of 4 animals progressed). However, no disease was detected in any of the mice belonging to the NALM6 + ARI-0001 group (0 of 6 mice progressed). The experiment was completed and the animals sacrificed at days 16–17, when the animals belonging to the NALM6 group started to show evident signs of disease. To confirm bioluminescence imaging data, bone marrow and blood cells were processed for flow cytometry. Anti-human CD19 staining confirmed the presence of tumor cells in the NALM6 and NALM6 + UT groups, while no significant percentages of tumor cells were detected in the other groups (Figures 2B and 2C) compared to control.

### Comparison of Cytotoxic Activity of ARI-0001 Cells to Other CART19 Constructs

**Figure 3:**
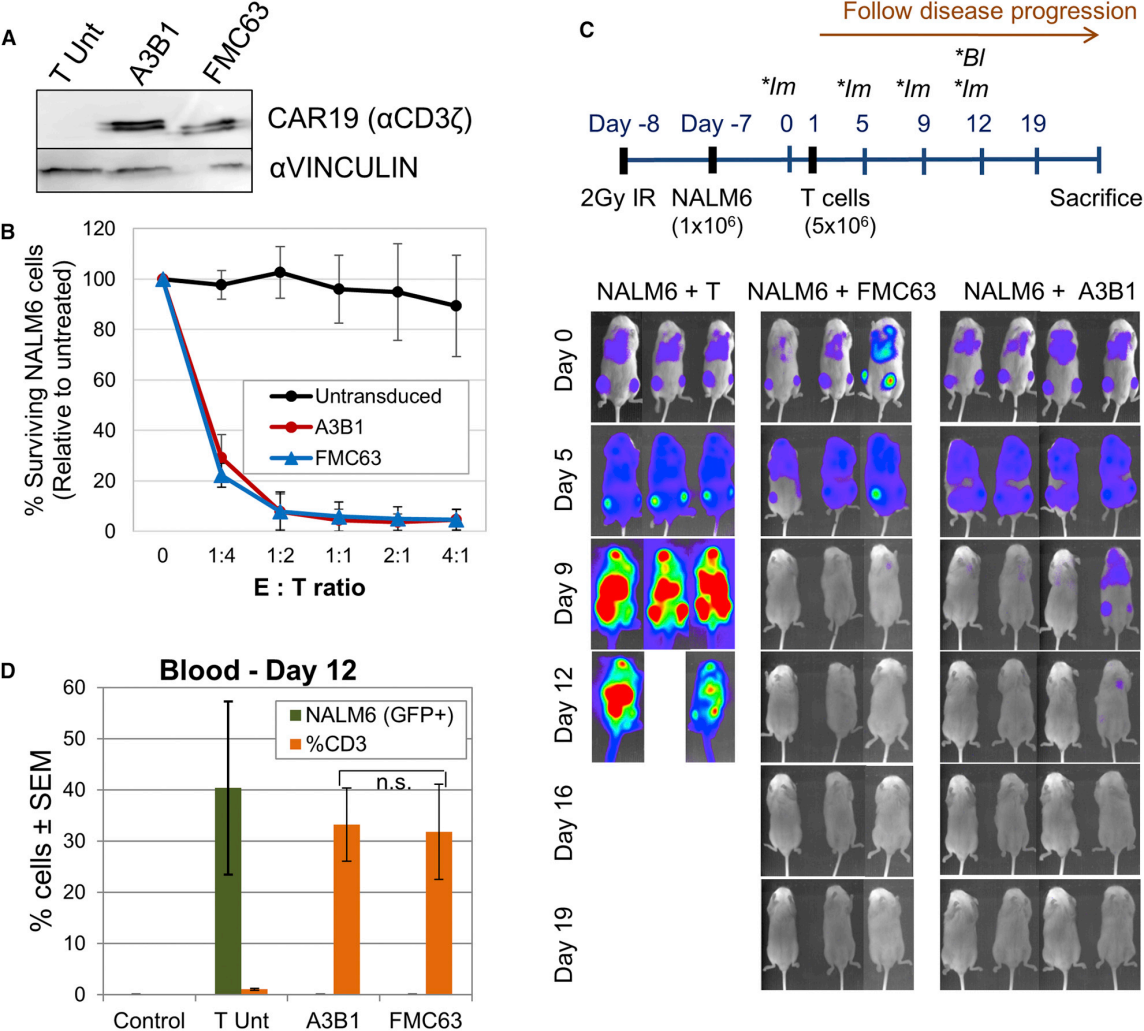
Comparison of Anti-tumor Activity of A3B1- and FMC63-based CAR T Cells (A) Detection of chimeric antigen receptor (CAR) expression by western blot. (B) Cytotoxicity assay of CART19 cells versus NALM6 cells after 4 hr of co-culture. Percent target surviving cells, relative to untreated, is shown (mean of 3 experiments ± SEM). (C) Upper panel shows a timeline of experimental design (*Im, bioluminescent image; *Bl, blood sample). Lower panels show bioluminescence images showing disease progression at different days. (D) Detection of tumor (GFP+) cells and CD3+ cells in blood by flow cytometry. Control, control mice (no tumor or T cells were injected).

To investigate how ARI-0001 cells, which contain the scFv from the A3B1 antibody, perform compared to other CART19 cells currently in use for the treatment of CD19+ malignancies, we cloned the scFv of the FMC63 antibody in our vector. The rest of the CAR construct remained the same, so we could directly compare the efficiency of both scFv fragments. For these analyses, we transduced PBMCs with a lentivirus containing each one of the CAR constructs. CAR19 protein expression was confirmed by western blot (Figure 3A). The cytotoxic activity of A3B1 and FMC63 CAR T cells was then compared *in vitro*. Figure 3B demonstrates no significant difference in cytotoxic potency between the CARs.

The two CAR constructs were then compared *in vivo*. For this experiment, the disease was allowed to progress before administering CAR T cells. The complete experimental design is depicted in Figure 3C. As shown in Figure 3C, NALM6 cell engraftment was detected by bioluminescence imaging 6 days after injecting NALM6 cells in all mice. Following the administration of UT T cells, disease continued to progress in the NALM6 + T group, while A3B1 and FMC63 CAR T cells progressively eliminated the tumor. Animals in the NALM6 + T group were sacrificed between days 11 and 12 due to disease progression. Quantification of the bioluminescence images indicated no statistical difference in the capacity of A3B1 and FMC63 CAR T cells to eliminate the tumor cells (Figure S4). Finally, flow cytometry performed on blood of these mice, taken at day 12 after T cell injection, confirmed no detectable disease in the blood of treated mice and T cell expansion (Figure 3D). The level of disease eradication and T cell expansion was equal between the two groups (A3B1 and FMC63).

Altogether, these data indicate that A3B1 scFv is capable of binding to CD19, triggering a cytotoxic response, and eliminating a tumor to a level similar to FMC63.

### Large-Scale CAR19 Lentivirus Production

Having demonstrated the efficacy and specificity of ARI-0001 cells *in vivo* and *in vitro*, we proceeded to set up and standardize the conditions for patient-scale ARI-0001 cell production.

**Figure 4:**
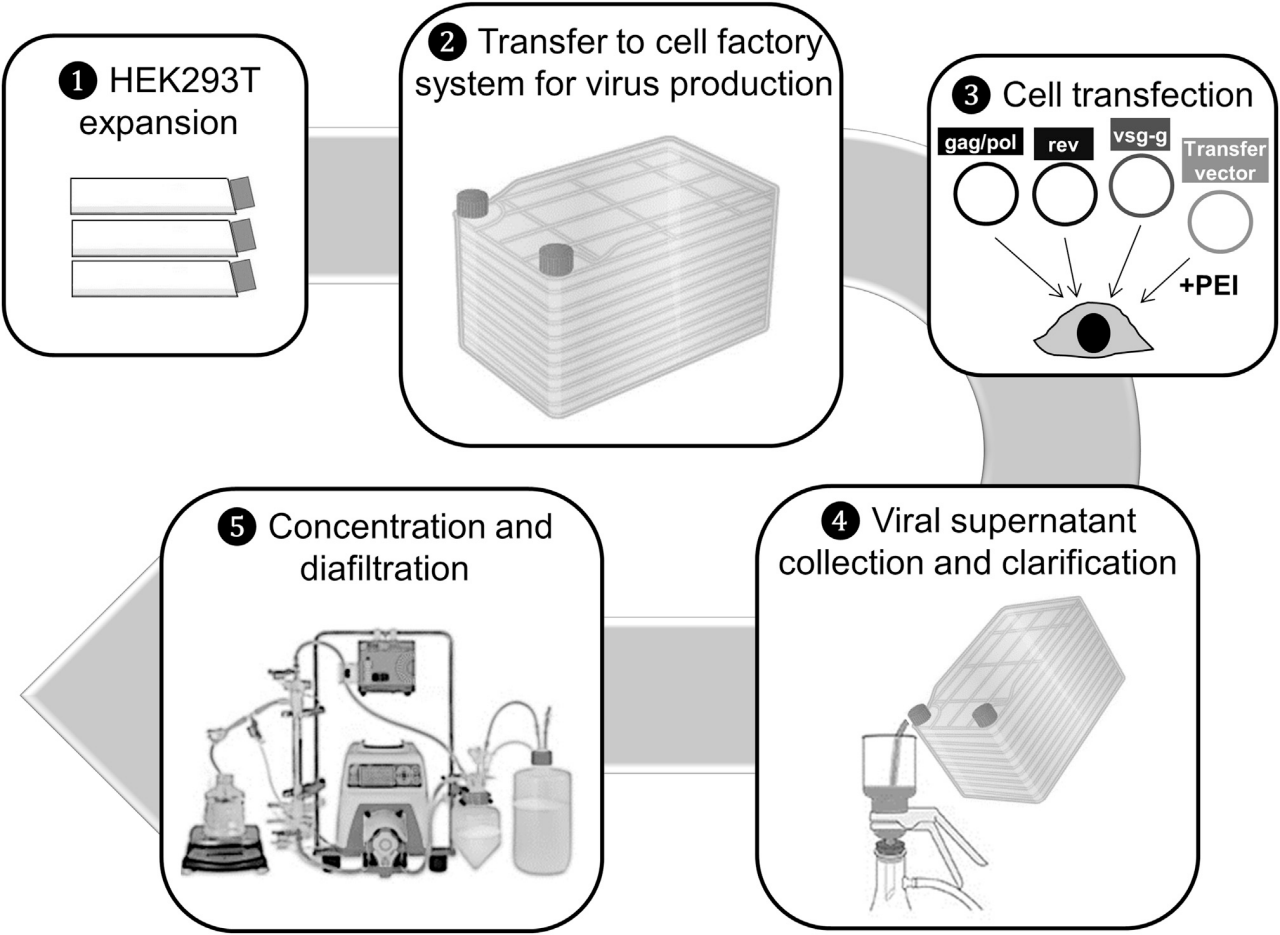
Diagram Depicting the Main Steps of Large-Scale CAR19 Lentivirus Production as Currently Established at Hospital Clínic de Barcelona

To produce enough lentiviral supernatant to complete the clinical trial, we scaled up our virus production method, and we conducted the entire process inside a clean room facility following Good Manufacturing Practice (GMP) guidelines, although lentiviral supernatant was considered to be an intermediate reagent in terms of drug agency approval. An overview of the process highlighting the critical steps is depicted in Figure 4. Each lot consisted of 4 L of unconcentrated virus and the production time per lot was 12 days. HEK293T was used as the packaging cell line. Before starting production, HEK293T master and working cell banks were prepared, so all lots were produced using HEK293T from the same passage.

For each production, we first expanded HEK293T in T175 flasks for 2 passages (expanding from 80 × 10^6^ cells to a minimum of 2,829 × 10^6^ cells). Cells were then transferred to four 10-layer CellStacks cell culture chambers (Corning) and one 1-layer CellStack to control for cell proliferation. Plasmid transfection was carried out the next day using 3.86 mg polyethylenimine (PEI), 763 μg transfer vector, 377 μg pMDLg-pRRE, 188 μg pRSV-Rev, and 221 μg pMD2.G per liter. Viral supernatants were collected 2 days later and clarified using a 0.45-μm polyvinylidene fluoride (PVDF) membrane. 4 L viral supernatant was finally concentrated and diafiltered using KrosFlo Research II*i* Tangential Flow Filtration System (Spectrum Labs) and 500 kD modified polyethersulfone (mPES) hollow fibers. 2 L PBS was used as diafiltration buffer. Each lot was concentrated to 100 mL, aliquoted in 10-mL bags, and kept at −80°C until use. Smaller aliquots were also kept for viral titer determination and sterility and purity analyses. For protocol validation, 3 viral lots were produced and analyzed. The results of analyses performed on these 3 lots are shown in Table 1. Viral titer of frozen-concentrated virus ranged between 1.1 and 2.2 × 10^8^ transducing units (TU)/mL. Quality control testing indicated that all three lots were negative for bacterial-fungal growth, mycoplasma, or replication-competent lentivirus (RCL). Virus identity was also confirmed by PCR amplification of principal virus components.

**Table 1:** Results and Quality Controls of GMP-Grade Viral Productions of 3 Supernatant Lots

Parameter	Method	Acceptance Criteria	Lot 1	Lot 2	Lot 3
Appearance	visual inspection	yellowish liquid solution	cloudy liquid solution	cloudy liquid solution	cloudy liquid solution
Viral titer	limiting dilution	>3.75 × 10^7^ TU/mL	2.29 × 10^8^ TU/mL	1.68 × 10^8^ TU/mL	1.10 × 10^8^ TU/mL
Sterility	microbial growth	sterile	sterile	sterile	sterile
Mycoplasma	PCR	absent	absent	absent	absent
Identity	PCR	positive	positive	positive	positive
RCL (replication-competent lentivirus)	real-time PCR	absent	absent	absent	absent

### ARI-0001 Cell Production

ARI-0001 cells were produced using CliniMACS Prodigy (Miltenyi Biotec). Apheresis products were subjected to CD4- and CD8-positive selection, and 100 × 10^6^ T cells were then cultured and activated using anti-CD3 and anti-CD28 antibodies. At 24 hr after activation, cells were transduced with CAR19 lentivirus (MOI = 10). Cells were cultured in media containing IL-7 and IL-15 until the desired cell number was reached (typically 8–9 days). T cell expansion with IL-7 and IL-15 has been shown to result in a much more T_N_ and T_SCM_ phenotype than when using expansion with IL-2, which favors T_EFF_.^16^ The product was collected in NaCl 0.9% + 0.5% human serum albumin (HSA). To test the consistency and robustness of our production method, we conducted three procedures using apheresis products from three different healthy donors. Our goal was to reach a minimum of 35 × 10^6^ ARI-0001 cells and ≥20% transduction efficiency.

**Figure 5:**
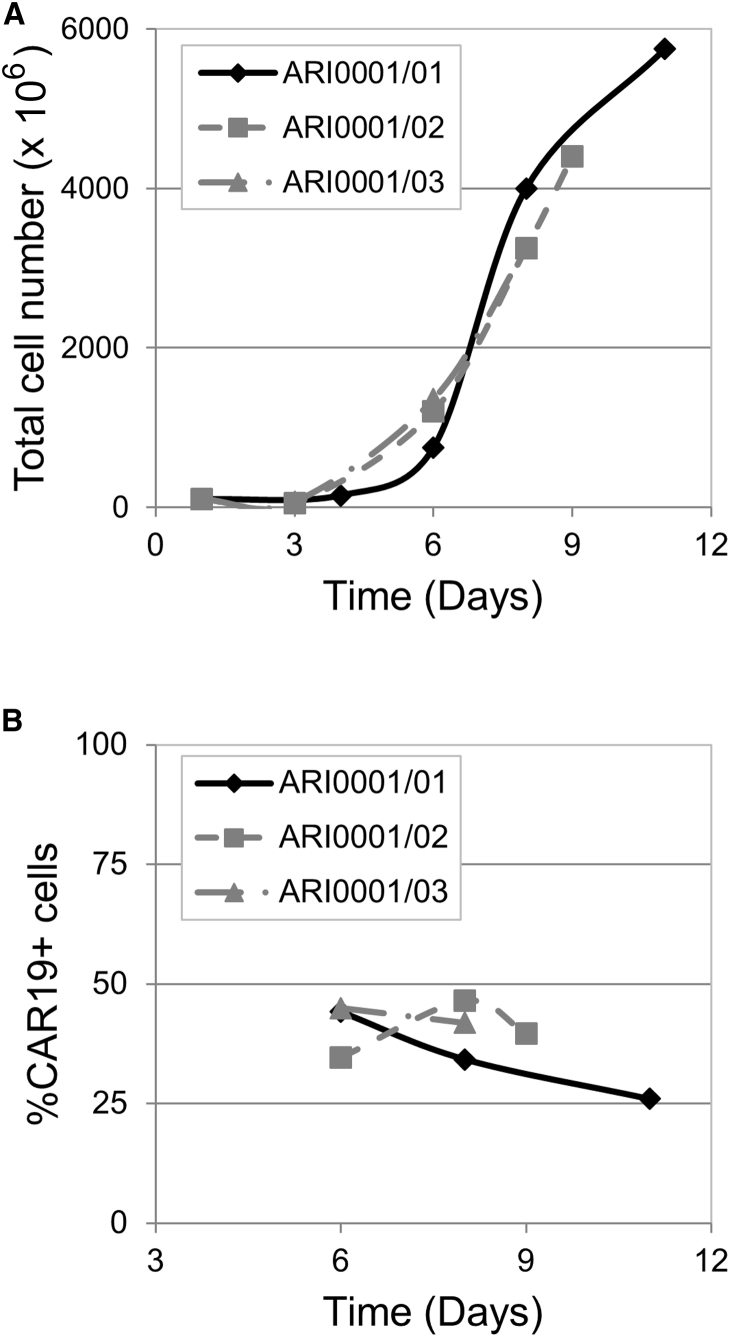
Results of 3 Validation Processes of ARI-0001 Cell Production Using Healthy Donors (A) Total cell number at different time points. (B) Percentage of CAR19-expressing cells at different time points.

As shown in Figure 5, the expansion time varied between 8 and 11 days. Run ARI0001/01 was allowed to proceed to day 11 to test Prodigy’s T cell expansion capacity, but the rest of the runs were stopped earlier (day 9 and day 8, respectively), since the minimum number of cells required was already reached. A mean of 3,780 × 10^6^ total cells was obtained, and the percentage of transduction averaged 35.8% at the time the cell expansion was terminated. Therefore, the acceptance criteria were reached in all three procedures. A full list of the quality tests conducted on the final products and the acceptance criteria that were defined for each of them is provided in Table 2. As shown in the same table, all ARI-0001 products obtained met the acceptance criteria established for all parameters regarding purity, safety, and potency. The results of *in vitro* cytotoxicity assay (potency) performed with the ARI-0001 final products are shown in Figure S5.

**Table 2:** ARI-0001 Product Specification List and Acceptance Criteria

Parameter	Method	Acceptance Criteria	ARI0001/01	ARI0001/02	ARI0001/03
Appearance	visual inspection	cloudy liquid solution	cloudy liquid solution	cloudy liquid solution	cloudy liquid solution
Number of CAR19+ cells	Neubauer cell counting and flow cytometry	35 × 10^6^ cells for a 70-kg patient (>0.5 × 10^6^ cells/kg)	1,495 × 10^6^	1,746 × 10^6^	1,340 × 10^6^
CAR19+ cells (%)	flow cytometry	≥20%	26.0	41.9	39.7
CD3+ cells (%)	flow cytometry	≥70%	97.9	98.6	98.6
Cell viability (%)	Neubauer cell counting with trypan blue exclusion	≥70%	97.9	98.1	98.0
Sterility	microbial growth	sterile	sterile	sterile	sterile
Mycoplasma	PCR	absent	absent	absent	absent
Endotoxin	chromogenic assay	≤0.5 EU/mL	≤0.5 EU/mL	≤0.5 EU/mL	≤0.5 EU/mL
Adventitious viruses	PCR	absent	absent	absent	absent
Number of transgene copies/cell	real-time PCR	≤10 copies/cell	1.45	2.32	2.51
RCL (replication-competent lentivirus)	real-time PCR	absent	absent	absent	absent
Cytotoxic potency	flow cytometry	(a) NALM6 cell surviving fraction using CART19 ratio E:T 1:1 <70% or (b) difference in NALM6 cell surviving fraction between CART19 versus untransduced T using ratio E:T 4:1 >50%	(a) 14.9; (b) 90.9	(a) 40.4; (b) 64.6	(a) 43.0; (b) 67.0

## Discussion

The use of genetically modified autologous T cells that express an anti-CD19 CAR (CART19) has shown impressive response rates for the treatment of CD19-positive B cell malignancies in several clinical trials. However, bringing this experimental treatment to commercialization phase is a lengthy process. The urgent need to make this treatment available to our patients prompted us to develop a new anti-CD19 CAR based on our own anti-CD19 antibody (A3B1).

There are many different CART19 constructs currently being evaluated in pre-clinical as well as in clinical studies.^12^ We decided to use a second-generation CAR with 4-1BB co-stimulator, based on the CAR developed by UPenn-CHOP, since it has shown a significantly longer persistence of CART19 cells both in mice and in humans.^17^, ^18^ We saw that our A3B1-CART (ARI-0001) cells were able to efficiently and specifically eliminate CD19-positive cells, both *in vitro* and *in vivo*, triggering a pro-inflammatory cytokine response and T cell proliferation—all necessary events for clinical efficacy. Our construct incorporates the scFv belonging to the A3B1 anti-CD19 antibody, which has never been used for therapeutic purposes before. We demonstrated that the *in vitro* and *in vivo* efficacy of ARI-0001 cells was similar to other constructs currently in use. This indicates that A3B1 antibody has a good avidity for its epitope and is consistent with the fact that CD19 possesses a single dominant epitope or adjacent epitopes.^19^ Thus, a change of scFv might not be as determinant for a good CAR19 response as with other target proteins.

Having shown that ARI-0001 cells perform as expected in pre-clinical studies and their *in vitro* effectivity might be comparable to other CART19 constructs currently used, the next step was to set up the infrastructure and the procedures to be able to move ARI-0001 cells to the clinic. This represents a considerably big enterprise for a relatively small publicly funded institution, but its success relies on two important facts: (1) involvement of a large number of groups from different disciplines and organizations in the project, which included basic scientists, hemato-oncology and immunotherapy clinical units, and GMP facilities with expertise in cellular therapies; and (2) CARs other than anti-CD19 that are currently being developed easily at the pre-clinical stage by several basic science labs in the Hospital Clínic-influenced area. Therefore, the platform created to transfer the anti-CD19 CAR from bench to bedside will also serve to promote quicker and easier transfer of other CARs to clinic.

ARI-0001 cell production includes two independent procedures: (1) large-scale vector production, and (2) *ex vivo* transduction and expansion of genetically engineered T cells. For vector production, we chose a third-generation lentiviral vector, since it is considered to be safer for patients than retroviral vectors or second-generation lentivirus.^20^, ^21^, ^22^ However, it is important to note that a third-generation lentiviral system is the least efficient of the three, in terms of quantity of viral particles per milliliter obtained, thus increasing the cost of the process. Large-scale lentivirus production can constitute a bottleneck of CAR T cell production. Many different cell culture systems can be used, from regular T175 flasks for adherent cultures to bioreactors. We decided to use an intermediate system, similar to a previously described method,^23^, ^24^ consisting of expanding HEK293T in T175 and then transferring to ten-layer flasks with 1-L capacity each for cell transfection and viral supernatant collection. We produced viral batches of 4-L unconcentrated virus each, which allowed the transduction of 8 patients/viral batch. The viral titer obtained was in range with what has been reported by other viral production facilities, reviewed in Merten et al.^25^ There is an obvious limit in the capacity of scaling up this method. Therefore, depending on the number of patients that are planned to be treated per year, other systems, including the use of bioreactors and cell suspension systems, should be considered.^26^, ^27^

There are many different systems currently being used for the *ex vivo* transduction and expansion of CAR T cells.^28^, ^29^ We decided to use the CliniMACS Prodigy system for the production of ARI-0001 cells, since it offers two key advantages that fitted well with our production model: (1) CliniMACS Prodigy is a semi-automated system that allows magnetic cell separation, cell activation, transduction, and expansion in a single machine; and (2) it is a closed GMP-compliant system, bypassing the need to conduct the whole process inside a specific GMP gene therapy clean room. Although the CliniMACS Prodigy also has some disadvantages compared to other technologies available, i.e., WAVE expansion system allows for a significantly higher T cell expansion and process adaptability,^30^ it was shown to be a robust and reproducible procedure in our hands.

In conclusion, we have developed a novel anti-CD19 CAR construct based on a well-defined design (the UPenn-CHOP proposal), conformed by an scFv of our own anti-CD19 mAb (A3B1) plus the 4-1BB- and CD3z-signaling domains. After positively fulfilling pre-clinical experiments and demonstrating robust and reproducible lentivirus and ARI-0001 cell production, a pilot clinical trial (CART19-BE-01, ClinicalTrials.gov: NCT03144583) for the treatment of CD19-positive malignancies was recently launched in 2017 at Hospital Clínic and Hospital Sant Joan de Déu in Barcelona (Spain) and is now ongoing. Current results of more than 10 ARI-0001 clinical grade products, developed for patients enrolled in ClinicalTrials.gov: NCT03144583, show characteristics of equivalence to the products presented here corresponding to the validation step (unpublished data). Taking all these data together, this work demonstrates that ARI-0001 cell production is robust, reproducible, and affordable for academic institutions that try to introduce this effective immunotherapy to their patients.

## Materials and Methods

### Donors, Cell Lines, and A3B1 Anti-CD19 mAb

All protocols were approved by the corresponding Institutional Review Board. Healthy donor blood buffy-coats were obtained from the local reference blood bank (Banc de Sang i Teixits, Barcelona). NALM6, HL60, K562, and 300.19 cell lines were purchased from the American Type Culture Collection (ATCC). These four cell lines were cultured in RPMI media + 10% fetal bovine serum (FBS) + antibiotics. The HEK293T cell line was also purchased from the ATCC (CRL-11268) and cultured in DMEM + 10% FBS + antibiotics. All cell lines were grown at 37°C and 5% CO_2_. 300.19-hCD19 stable transfected cells were produced using pUNO1-CD19 cDNA (InvivoGen, San Diego, CA), as described in Massaguer et al.^31^

The A3B1 murine anti-CD19 mAb was generated at the Department of Immunology (Hospital Clínic de Barcelona), and its anti-CD19 specificity was confirmed by our own recent (Figure 1) and previous studies^15^ and several commercial distributors (Abcam, Immunostep, and CliniSciences). Studies of A3B1 reactivity and blocking experiment were performed as described in Romero et al.^32^

### CAR19 Cloning and Lentivirus Production

The sequence corresponding to the variable light (VL) and variable heavy (VH) regions of A3B1 antibody was extracted from A3B1 hybridoma cells using Mouse Ig-Primer Set (Novagen, 69831-3). The complete CAR19 sequence (including signal peptide, A3B1 scFv, CD8 hinge, and transmembrane regions 4-1BB and CD3z) was synthesized by GenScript and cloned into the third-generation lentiviral vector pCCL (kindly provided by Dr. Luigi Naldini; San Raffaele Hospital, Milan),^20^ under the control of EF1α promoter (Figure 2A). The scFv sequence of the FMC63 antibody was extracted from patent WO2012079000^33^ and similarly cloned into the pCCL vector.

To produce lentiviral particles for pre-clinical studies, HEK293T cells were transfected with our transfer vector (pCCL-EF1α-CAR19) together with packaging plasmids pMDLg-pRRE (Addgene, 12251), pRSV-Rev (Addgene, 12253), and envelope plasmid pMD2.G (Addgene, 12259), using linear PEI molecular weight (MW) 25,000 (Polysciences, 23966-1). Briefly, 6 × 10^6^ HEK293T cells were plated 24 hr before transfection in 10-cm dishes. At the time of transfection, 14 μg total DNA (6.9 μg transfer vector, 3.41 μg pMDLg/pRRE, 1.7 μg pRSV-Rev, and 2 μg pMD2.G) was diluted in serum-free DMEM. 35 μg PEI was added to the mix and incubated for 20 min at room temperature. After incubation, DNA-PEI complexes were added onto the cells cultured in 7 mL complete DMEM. Media were replaced 4 hr later. Viral supernatants were collected 48 hr later and clarified by centrifugation and filtration using a 0.45-μm filter. Viral supernatants were concentrated using ultracentrifugation at 26,000 rpm for 2 hr 30 min. Virus-containing pellets were resuspended in complete XVivo15 media and stored at −80°C until use.

### Lentivirus Titration

The number of transducing units (TU/mL) was determined by the limiting dilution method. Briefly, HEK293T cells were seeded 24 hr before transduction. Then, 1:10 dilutions of the viral supernatant were prepared and added on top of the cells in complete DMEM + 5 μg/mL Polybrene. Cells were trypsinyzed 48 hr later and labeled with allophycocyanin (APC)-conjugated AffiniPureF(ab’)_2_ Fragment Goat anti-mouse immunoglobulin G (IgG) (Jackson ImmunoResearch Laboratories, 115-136-072) before being analyzed by flow cytometry. A dilution corresponding to 2%–20% of positive cells was used to calculate viral titer.

### T Cell Transduction and Culture Conditions

Healthy donor PBMCs were obtained from buffy-coats by density-gradient centrifugation (Ficoll) after donation was consented, following the instructions of the Ethics Committee. While monocytes were eliminated by conventional plate adhesion, the remaining cells were cultured in X-VIVO 15 Cell Medium (Cultek, BE02-060Q), 5% AB human serum (Sigma, H4522), penicillin-streptomycin (100 μg/mL), and IL-2 (50 IU/mL; Miltenyi Biotec). Cells were then activated and expanded for 24 hr using beads conjugated with CD3 and CD28 mAbs (Dynabeads, Gibco, 11131D), and they were transduced 24 hr later with the lentivirus by overnight incubation in the presence of polybrene (Santa Cruz Biotechnology, sc-134220) at 8 μg/mL. A period of cell expansion of 6–8 days was necessary before conducting experiments. Three different cell transductions using three different PBMC donors were used to conduct the experiments in triplicate.

### Flow Cytometry

The following mAbs against human proteins, all from BD Biosciences, were used: CD3-fluorescein isothiocyanate (FITC), CD4-BV421, CD8-APC, CD19-phycoerythrin (PE), and CD33-PE. 7-AAD was used as a viability marker (Thermo Fisher Scientific, A1310). CAR19 expression was detected with an APC-conjugated AffiniPureF(ab’)_2_-fragment goat-anti-mouse IgG (Jackson ImmunoResearch Laboratories, 115-136-072). Samples were run through the fluorescence-activated cell sorting flow cytometer BD FACSCanto II (BD Biosciences), and data were analyzed using the BD FACSDiva Software.

### *In Vitro* Assays of Anti-tumor Efficacy

CART19 (ARI-0001) or UT T cells were co-cultured for 16 hr, unless otherwise indicated, with tumor target cell lines (NALM6 or HL60) or primary B-ALL tumor cells, at different E:T ratios, maintaining a fixed number of target cells. Then, cells were transferred to TruCOUNT tubes (Becton Dickinson, 340334) and incubated with mAbs against human CD4, CD8, CD19 (or CD33), and 7-AAD. Cytotoxicity was determined by calculating the number of surviving target cells (identified as 7-AAD-negative and CD19- or CD33-positive cells, for NALM6 and HL60 target cells, respectively). Acquisition was stopped after a fixed number of beads was acquired and absolute cell numbers were calculated, allowing comparison between different E:T ratios. Co-culture supernatants were analyzed for cytokine production (IFNγ, TNF-α, and IL-10) by ELISA, following the manufacturer’s instructions (Becton Dickinson OptEIA). All experiments were run in triplicate.

ARI-0001 cell proliferation in response to CD19 antigen was measured using a CFSE assay. Briefly, ARI-0001 cells were labeled with 1 μM CFSE, washed, and cultured with or without proliferating stimuli (IL-2 50 U/mL, NALM6 or K562 cells). NALM6 or K562 cells were added at an E:T ratio = 1:1. Assay was stopped after 96 hr and cells were stained with anti-CD4 and anti-CD8. CFSE staining was measured in CD4+ and CD8+ cells, and proliferating index (PI) was calculated (PI = sum of number of cells in different generations/calculated number of original cells).

### *In Vivo* Xenograft Model of Anti-tumor Efficacy and Safety

Animal studies were approved by CEEA-UB, the competent ethics committee for animal experimentation.

For the first experiment, 3-month-old NSG mice were infused intravenously (tail vein) with NALM6 tumor cells (1 × 10^6^ cells/mice) expressing GFP and luciferase. Mice were then randomly allocated to ARI-0001 cells (10 × 10^6^/mice), UT cells (10 × 10^6^ cells/mice), or vehicle.

ARI-0001 or UT cells were infused 3 days after the infusion of NALM6. Tumor growth was evaluated weekly by bioluminescence imaging (Hamamatsu detector) after the intravenous administration of D-luciferin. Mice were sacrificed at days 16–17, and tumor burden was measured in blood and bone marrow samples by flow cytometry.

The second experiment was designed to compare the two CAR19 constructs (A3B1 and FMC63). The 3-month-old NSG mice were irradiated with 2 Gy at day −8. NALM6 tumor cells (1 × 10^6^ cells/mice) expressing GFP and luciferase were infused intravenously (tail vein) the following day, except in one mouse (control mouse). Tumor cells were allowed to engraft for several days. Mice were randomly allocated to NALM6 + T (n = 3), NALM6 + A3B1 (n = 4), and NALM6 + FMC63 (n = 3) groups. 5 × 10^6^ cells/mice were infused intravenously (UT T cells, T A3B1, or T FMC63). Bioluminescence images were taken twice a week to follow disease progression. A blood sample was taken at day 12 to measure disease burden and T cell expansion by flow cytometry. Bioluminescence images were quantified using ImageJ.

### Patient-Scale ARI-0001 Cell Production

Leukocytapheresis from healthy donors was obtained in the Apheresis Unit at the Hospital Clínic de Barcelona with informed consent and approved by the Ethics Committee of our hospital. Apheresis procedures were performed using the Amicus device (Fresenius Kabi, Lake Zurich, IL). A minimum of 1 × 10^8^ T cells diluted in 50 mL plasma was required. Cells were cultured in the CliniMACS Prodigy system (Miltenyi Biotec) using TexMACS media supplemented with 3% human AB serum and with IL-7 and IL-15 (Miltenyi Biotec 170-076-111 and 170-076-114, respectively). For T cell activation, TransACT GMP Grade (Miltenyi Biotec, 170-076-156) was used.

## Author Contributions

M. Castella and A.B. designed and performed experiments, analyzed data, and wrote the manuscript. R.M.-I., J.T., A.V., J.M.C., E.T., P.P.-G., and J. Cid supervised specific parts of the study. V.R., G.S., L.P.-A., B.M.-A., M. Caballero, B.M., J. Castaño, C.B., O.B., E.A.G.-N., and M.L. performed experiments and procedures. C.S.-P., P.E., and R.V. provided key reagents. D.B.-R., V.O.-M., T.B., E.C., and P.M. critically read and edited the manuscript. A.U.-I., J.Y., S.R., J.D., and M.J. coordinated and supervised the study.

## Conflicts of Interest

S.R. declares speaker’s bureau and travel expenses with the following companies: Novartis, Shire, JazzPharma, and Erytech. All other authors declare that they have no competing interests.

## Supplemental Information

Document S1. Supplemental Materials and Methods and Figures S1–S5

Document S2. Article plus Supplemental Information
