# Supplementary material for: Development of a Novel Anti-CD19 Chimeric Antigen Receptor: A Paradigm for an Affordable CAR T Cell Production at Academic Institutions
Source: Mol Ther Methods Clin Dev. 2018 Dec 6;12:134–44. doi: 10.1016/j.omtm.2018.11.010 (PMC6319086; doi:10.1016/j.omtm.2018.11.010)
Supplement: Document S1. Supplemental Materials and Methods and Figures S1–S5 [file mmc1.pdf]

OMTM, Volume *12*

Supplemental Information

Development of a Novel Anti-CD19 Chimeric Antigen Receptor: A Paradigm for an Affordable CAR T Cell Production at Academic Institutions

Maria Castella, Anna Boronat, Raquel Martín-Ibáñez, Vanina Rodríguez, Guillermo Suñé, Miguel Caballero, Berta Marzal, Lorena Pérez-Amill, Beatriz Martín-Antonio, Julio Castaño, Clara Bueno, Olga Balagué, Europa Azucena González-Navarro, Carles Serra-Pages, Pablo Engel, Ramon Vilella, Daniel Benitez-Ribas, Valentín Ortiz-Maldonado, Joan Cid, Jaime Tabera, Josep M. Canals, Miquel Lozano, Tycho Baumann, Anna Vilarrodona, Esteve Trias, Elías Campo, Pablo Menendez, Álvaro Urbano-Ispizua, Jordi Yagüe, Patricia Pérez-Galán, Susana Rives, Julio Delgado, and Manel Juan

Supplemental information

1. Supplemental figures
2. Supplemental Materials and methods

1. Supplemental figures

Figure S1. Anti-CD19 A3B1 antibody validation

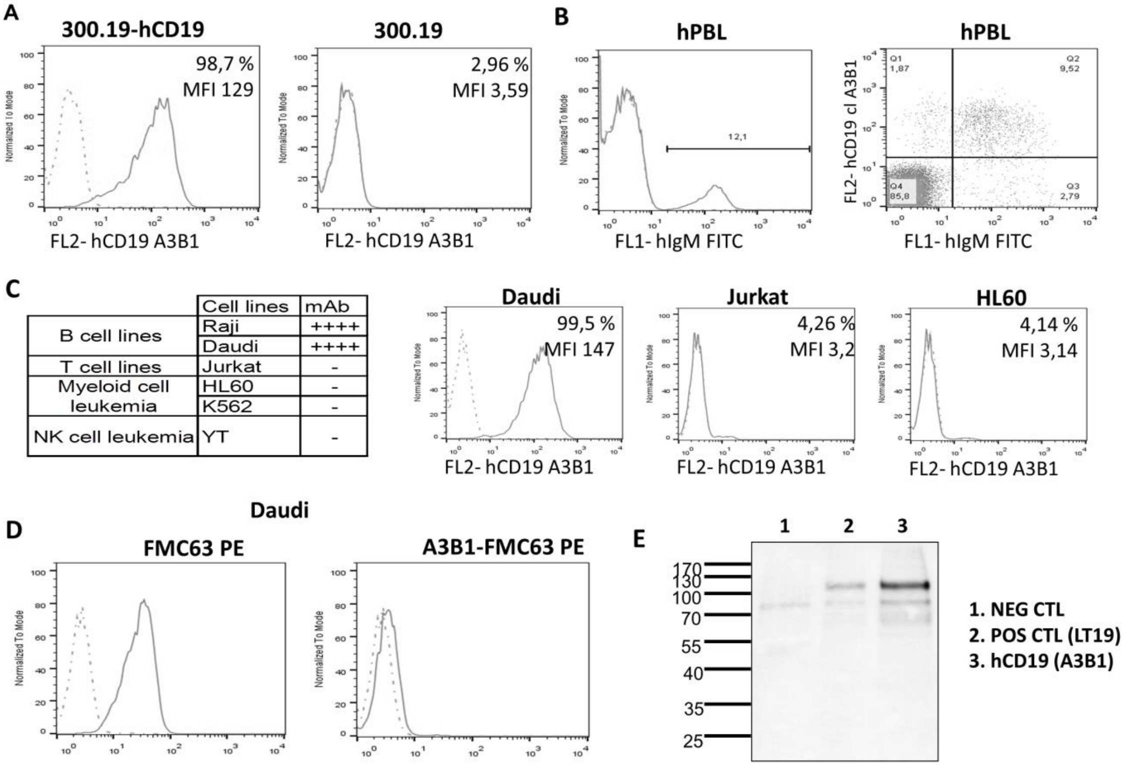

(A) Reactivity of mAb A3B1 with 300.19-hCD19 stable transfected cells and 300.19 untransfected cells. (B) Reactivity of mAb A3B1 with peripheral blood B cells, single staining and double staining with IgM. (C) Reactivity of mAb A3B1 with different hematopoietic cell lines: summary table (- = less than 20% positive cells; ++++ more than 90% of positive cells, and representative flow cytometry histograms with Daudi (B cell lines), Jurkat (T cell lines) and HL60 (myeloid cell lines). (D) Ability of mAb A3B1 to block the binding of CD19 FMC63 labeled antibody to DAUDI cell line, (E) Immunoprecipitation with mAb A3B1 from Daudi cell line.

Figure S2. *In vitro* evaluation of ARI-0001 cell cytotoxic activity

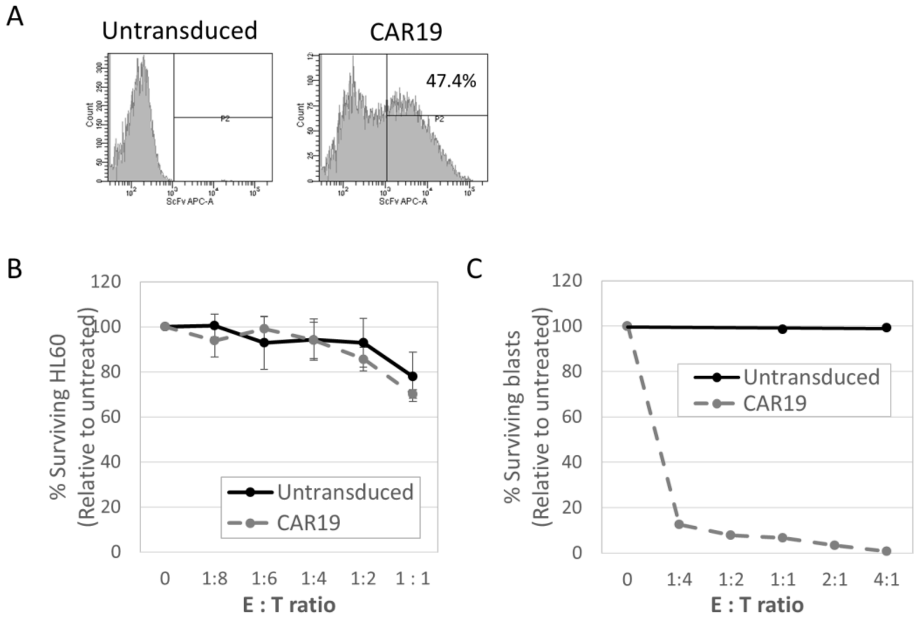

(A) Detection of CAR19 expression in T cells by flow cytometry. (B) Cytotoxicity assay of CART19 cells *vs* HL60 cells (CD19-) at 16h time-point. Percent target surviving cells, relative to untreated, is shown (Mean of 3 experiments ± SEM). (C) Cytotoxicity assay of CART19 cells *vs* a primary B-ALL at 16h time-point.

Figure S3. *In vitro* cytotoxicity assay – Flow cytometry gating strategy

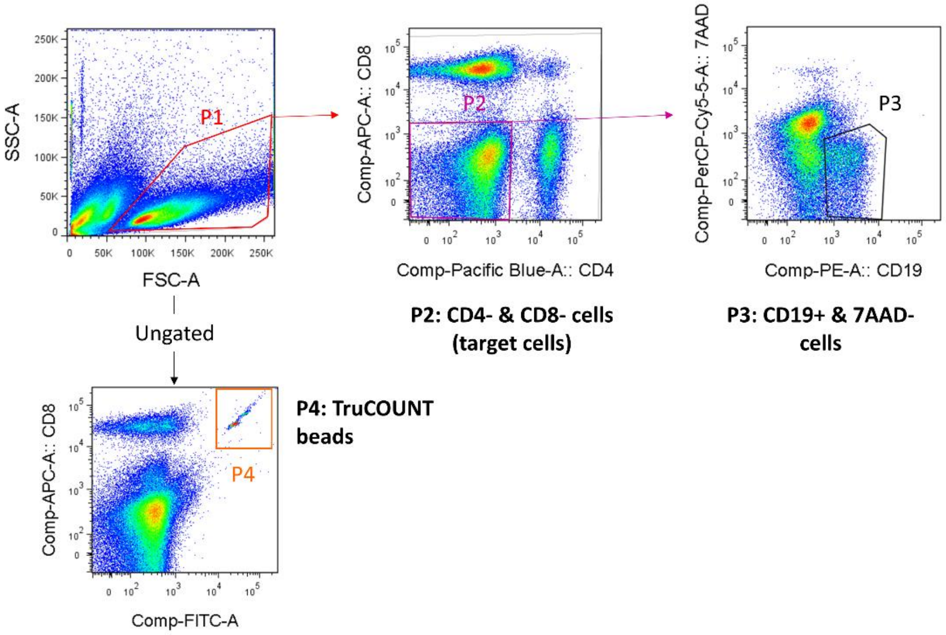

Flow cytometry gating strategy shown on a representative sample. Normal shaped cells are selected using P1 gate. To select target cells (NALM6) two consecutive gates are used. First, T cells are excluded first by gating CD4- and CD8- cells (P2). Then, alive NALM6 cells are selected by gating CD19+ and 7AAD- cells (P3). TruCOUNT beads are selected from ungated population (P4).

Figure S4. Quantification of tumor burden by bioluminescent imaging in a NALM6 xenograft mouse model, following T cell administration (Untransduced T cells, A3B1 or FMC63 CAR T cells).

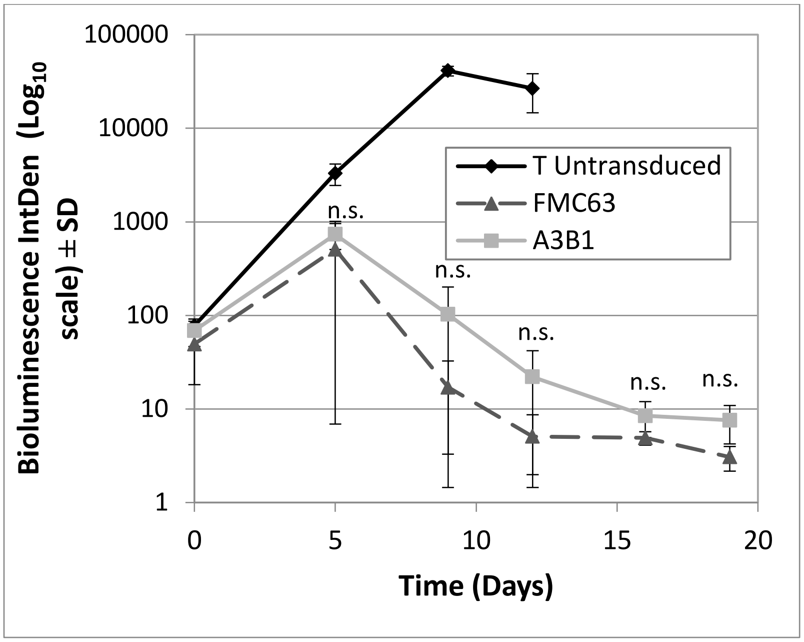

Bioluminiscent signal from each mouse at the different time points was quantified using Image J. Graphic shows how tumor signal increases in the T untransduced group and decreases in the CAR T cell treated groups. Mean IntDen±SD is shown. n..s. indicates no statistical difference between FMC63 and A3B1 gorups..

Figure S5. Cytotoxic activity of ARI-0001 cell products produced with CliniMACS Prodigy.

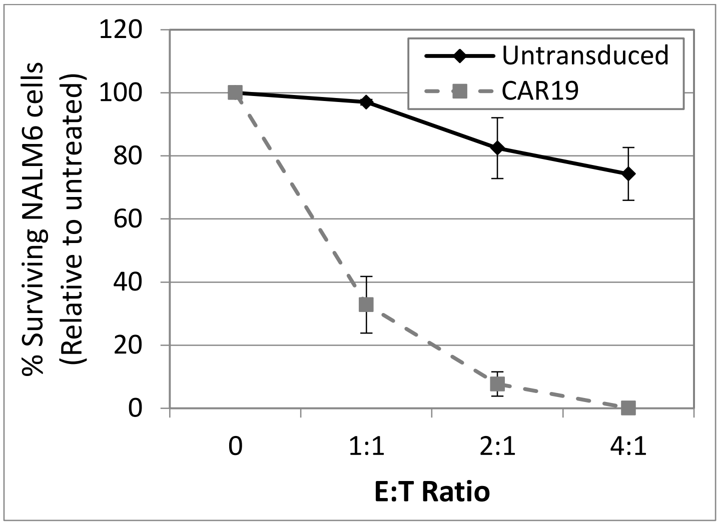

Cytotoxicity assay of ARI-0001 cell products produced with CliniMACS Prodigy, at 4h time-point. Percent target surviving cells, relative to untreated, is shown (Mean of 3 experiments ± SEM).

2. Supplemental Materials and methods

Immunoprecipitation and Western Blotting

Immunopreciptitation was performed as described in [19]. For regular western blotting, cells were lysed in 0.05 M Tris-HCl (pH 6.8), 2% SDS, 6% β-mercaptoethanol and boiled for 5min. SDS-PAGE electrophoresis was done using 12% acrylamide gels. Proteins were transferred to a PVDF membrane. Primary antibodies anti-CD3z (Santa Cruz Biotec, sc-166435) and anti-Vinculin (Abcam, ab129002) were diluted in blocking buffer (5% milk in TBS-Tween 20) and incubated overnight. Horseradish peroxidase—conjugated anti-mouse (Abcam) and anti-rabbit IgG (Cell Signalling) were used as secondary antibodies. Images were acquired using ImageQuant LAS4000 mini system (GE Healthcare).

Determination of number of transgene copies/cell

Number of transgene copies/cell was determined by quantitative real-time PCR, using Light Cycler® 480 SYBRGreen® I Master (Roche, Cat. N. 04707516001). Pairs of primers were designed against the GATA2 gene (control) and WPRE sequence (part of the transgene).

Primer sequences are as follows: GATA2_F: 5'tggcgcacaactacatggaa 3'; GATA2_R: 5'cgagtcgaggtgattgaagaaga 3'; WPRE_F: 5'gtcctttccatggctgctc 3'; WPRE_R: 5'ccgaagggacgtagcaga 3'. Absolute quantification method was used to determine copy number. Standard curves were prepared using 1:10 serial dilutions of plasmids containing GATA2 or transgene. Final number of molecules in the reaction ranged from 10^8 to 10^2 molecules. For GATA2 quantification, GATA2 cDNA was cloned in a pCRII-Topo vector (Invitrogen). pCCL-CAR19 vector was used in the same way to quantify transgene copy number. The following PCR program was used: 1) Initial denaturalization: 95°C, 5'; 2) 40 cycles of: 95°C, 10''; 58°C, 10''; 72°C, 5''; 3) Melting curve.

Detection of replication-competent lentivirus (RCL)

Presence of RCLs was evaluated using detection of VSV-G DNA in the final product as proposed by Sastry *et al.* 2003 [20]. Absolute quantification by real-time PCR using Light Cycler 480 SYBRGreen I Master was also used in this case. Primers used to detect VSV-G sequence are: VSVG-F: 5' tgcaaggaaagcattgaacaa 3' and VSVG-R: 5'gaggagtcacctggacaatcact 3'. The same quantification method and PCR program as for determination of transgene copies/cell was used.

Statistical analysis

Unless otherwise indicated, results are expressed as mean standard deviation of three independent experiments. P-values were determined by paired t test (when comparing two groups).
